# Supplementary material for: ZEB1‐mediated melanoma cell plasticity enhances resistance to MAPK inhibitors
Source: EMBO Mol Med. 2016 Sep 5;8(10):1143–61. doi: 10.15252/emmm.201505971 (PMC5048365; doi:10.15252/emmm.201505971)

Appendix Richard et al. (EMM-2015-05971)

- Appendix Supplementary Methods
- Appendix Supplementary Figure legends
- Appendix Supplementary Figures S1 to S9

Appendix Supplementary Methods

Q-PCR

The primers used in appendix figures were: human *Tyrosinase*, GCT GCC AAT TTC AGC TTT AGA and CCG CTA TCC CAG TAA GTG GA, human *E-cadherin*, CCC GGG ACA ACG TTT ATT AC and GCT GGC TCA AGT CAA AGT CC, human *Vimentin*, GAC CAG CTA ACC AAC GAC AAA and GAA GCA TCT CCT CCT GCA AT, human *SPARC*, CGA GCT GGA TGA GAA CAA CA and GTT GTC ATT GCT GCA CAC CT, human *AXL*, GGT GGC TGT GAA GAC GAT GA and CTC AGA TAC TCC ATG CCA CT, human *WNT5A*, GCC ATG AAG AAG TCC ATT GG and CTT CAA TTA CAA CCT GG CG.

Appendix Supplementary figure legends

Appendix Figure S1: *MITF, ZEB1, TWIST1* and *ZEB2* mRNA expression levels and IC50 for the BRAFi PLX4720 and the MEKi AZD6244 in *BRAF*^{V600}-mutated melanoma cell lines from the CCLE. IC50 (µM) are indicated. PLX4720 is an analog of PLX4032/vemurafenib. AZD6244/selumetinib is a MEKi for which clinical trials are still ongoing. Cell lines are ranked according to their PLX4032 IC50. Color code: IC50<5µM: green, IC50>5µM: red ; *MITF*, *ZEB1*, *TWIST1* and *ZEB2* expression levels: <7: green, >7: red.

Appendix Figure S2: *ZEB1* expression levels are higher in *BRAF*^{V600} or *NRAS*^{Q61R}-mutated melanomas compared to *BRAF/NRAS* WT tumors. Analyses of *ZEB1* expression according to the mutation status of *BRAF/NRAS* in the TCGA data set (n=341). (A) Whisker plot of *ZEB1* mRNA expression in *BRAF*^{V600}, *NRASQ61R* or *BRAF/NRAS* WT melanoma from the TCGA. (B) Histogram showing that 80% of ZEB1^{high} melanomas exhibit a *BRAF* or *NRAS* mutation, while 50% of ZEB1^{low} melanomas are *BRAF/NRAS* WT. Wilcoxon signed-rank test.

Appendix Figure S3: *ZEB1* overexpression in SKMEL5 melanoma cells induces switching towards a MITF^{low}/p75^{high} stem-like tumor initiating phenotype and promotes resistance to MAPKi (A) SKMEL5 cells were infected with retroviruses expressing *ZEB1*. Western blot analyses of ZEB1 and p75. GAPDH was used as a loading control. (B) Quantitative PCR analyses of *MITF* and *p75* upon *ZEB1* expression. mRNA expression levels are represented relatively to control cells (mean ± SD, n=3, Student's t-test). (C) Soft agar colony formation assay upon *ZEB1* expression. Scale bar = 200 µM. Histograms represent

quantitative analyses (mean ± SD, n=3). (D) FACS analyses of p75 expression upon *ZEB1* expression, after 10 days treatment with 300 nM PLX4032. Bar chart representing the mean percentage of p75-high, int, low and negative cells from 2 independent experiments (Fisher's Exact Test). (E) Clonogenic assay +/- PLX4032 (300 nM), +/- GDC-0973 (5 nM) treatment for 10 days. The graphs represent the mean number of colonies in 3 independent experiments. (Student's t test).

Appendix Figure S4: The levels of ZEB2 are decreased while the levels of invasion markers are induced in *ZEB1*-overexpressing A375 cells. (A) ZEB2 expression assessed by Western-blot and Q-PCR analyses upon *ZEB1* expression in A375 cells. GAPDH was used as a loading control. Of note, ZEB2 antibody also recognizes exogenously expressed ZEB1, which can be distinguished from endogenous ZEB2 by its higher molecular weight. (n=3, Student's t-test). (B) Q-PCR analyses of *Vimentin*, *SPARC*, *MMP1*, *AXL* and *WNT5A* upon *ZEB1* expression in A375 cells. mRNA expression levels are represented relatively to control cells (mean ± SD, n=3, Student's t-test). AXL was undetectable. n.s: non significant.

Appendix Figure S5: *p75* mRNA expression is positively correlated with *ZEB1* in human melanoma samples. Pearson correlation between *ZEB1* and *p75* mRNA expression in the TCGA data set (n=467).

Appendix Figure S6: *ZEB1* knock-down in A375 cells results in increased ZEB2 and E-cadherin expression levels. ZEB2 and E-cadherin expression levels were analyzed by western blot or Q-PCR upon *ZEB1* knock-down in A375 cells. mRNA expression levels are represented relatively to control cells (mean ± SD, n=3, Student's t-test).

Appendix Figure S7: PLX4032 IC50 values are not significantly modified upon *ZEB1* expression in A375, C-09.10 and GLO cells. Mean IC50 values (µM) from 3 experiments determined by ATP assays after 3 days of exposure to the drug.

Appendix Figure S8: AXL expression levels are increased in BRAFi resistant models. Western blot analyses of AXL in A375-R and SKMEL5-R *versus* the parental naive cells, and in GOKA and ESP cells. Actin was used as a loading control.

Appendix Figure S9: The ectopic expression of *TWIST1* in A375, SKMEL5 and 501MEL melanoma cells does not affect their colony forming capacity. A375, SKMEL5 and 501MEL cells were infected with retroviruses expressing *TWIST1*. Soft agar colony formation assays upon *TWIST1* expression showing no significant difference compared to control cells. (n=3, Student's t test). n.s: non significant.

Appendix Figure S1

Cell line	BRAF status	IC50 PLX4720	IC50 AZD6244	MITF	ZEB1	TWIST1	ZEB2
WM88	BRafV600	0,204	0,051	10,050	7,023	10,055	5,678
UACC62	BRafV600	0,250	0,035	9,496	6,795	9,947	7,957
A375	BRafV600	0,258	0,107	7,636	6,976	9,361	4,433
MALME3M	BRafV600	0,286	0,046	10,960	4,964	8,285	6,698
MELHO	BRafV600	0,313	0,108	11,149	4,937	7,993	5,733
SKMEL5	BRafV600	0,369	0,169	10,970	5,103	9,517	5,990
WM983B	BRafV600	0,511	0,088	10,053	4,524	10,098	7,070
COLO679	BRafV600	0,554	0,123	10,335	5,353	10,953	5,354
RVH421	BRafV600	0,771	0,257	10,445	6,511	8,977	5,936
IGR37	BRafV600	0,904	0,213	10,847	3,964	10,445	6,644
UACC257	BRafV600	1,064	0,234	10,996	4,321	7,833	7,957
WM1799	BRafV600	1,226	0,351	10,004	6,812	10,222	6,825
G361	BRafV600	1,287	0,419	10,831	4,805	10,403	7,216
HT144	BRafV600	1,336	0,374	9,477	6,528	9,514	7,239
WM2664	BRafV600	1,579	0,165	10,433	6,545	6,007	7,321
HS939T	BRafV600	2,103	0,494	9,724	5,563	10,040	6,841
K029AX	BRafV600	2,111	0,459	10,466	4,624	7,392	7,191
C32	BRafV600	2,207	1,360	9,984	5,881	9,861	6,251
MDAMB435S	BRafV600	2,313	0,422	7,947	6,302	9,300	6,642
COLO741	BRafV600	4,040	2,675	10,969	5,088	7,969	7,491
SKMEL24	BRafV600	5,155	2,313	7,357	6,612	8,475	7,358
HS695T	BRafV600	7,265	8	6,354	7,728	8,538	4,466
LOXIMVI	BRafV600	8	0,590	5,681	8,573	8,585	6,217
RPMI7951	BRafV600	8	8	6,201	9,207	10,103	5,820
WM793	BRafV600	8	8	6,389	6,674	9,797	5,408
WM115	BRafV600	8	8	6,847	6,618	10,725	6,859
IGR39	BRafV600	8	8	7,645	7,155	9,094	5,073
A2058	BRafV600	8	5,036	8,709	6,761	9,225	5,369

Appendix Figure S2

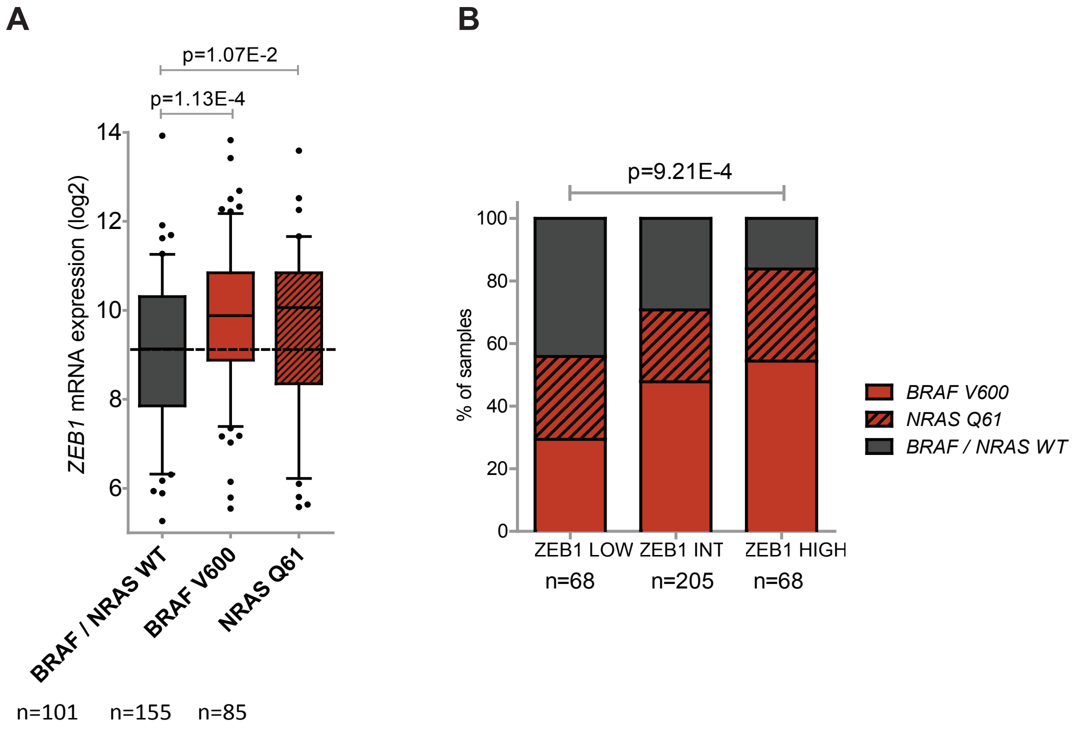

Appendix Figure S3

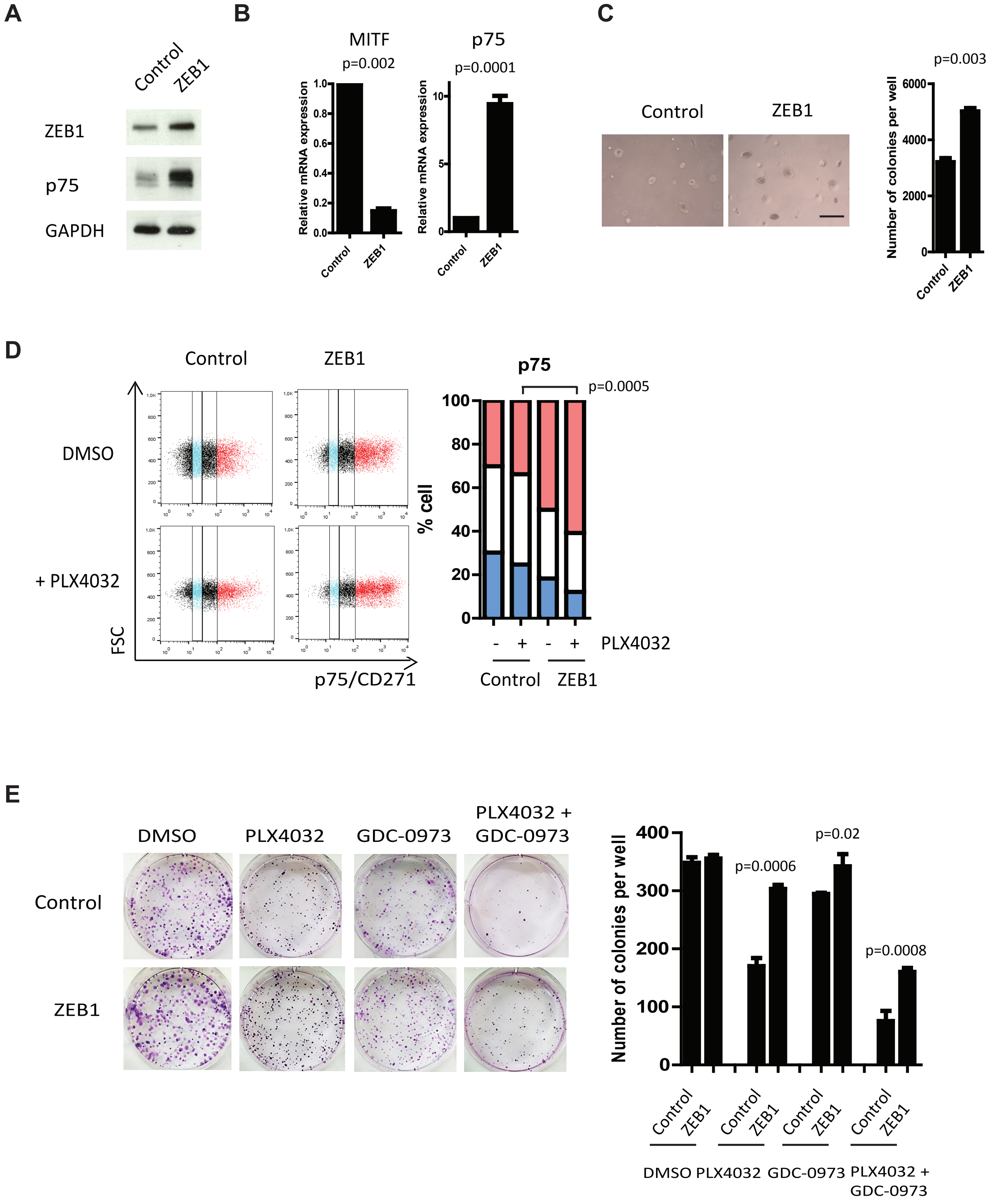

Appendix Figure S4

A

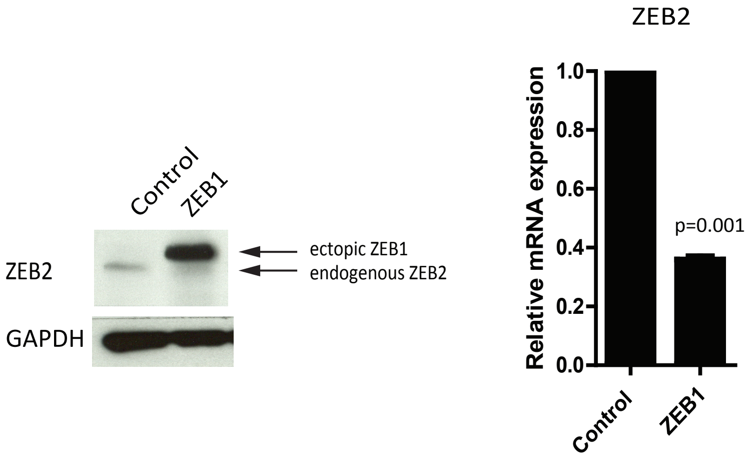

B

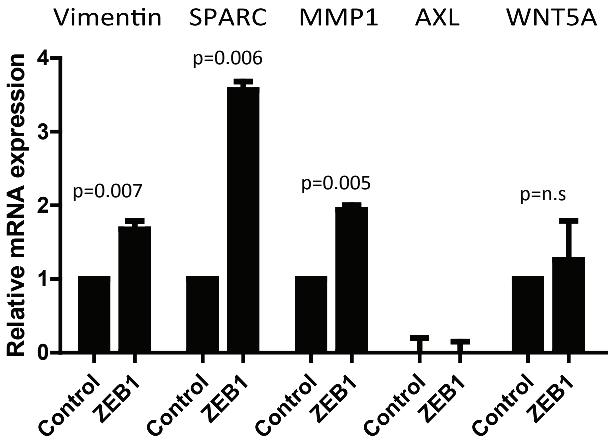

Appendix Figure S5

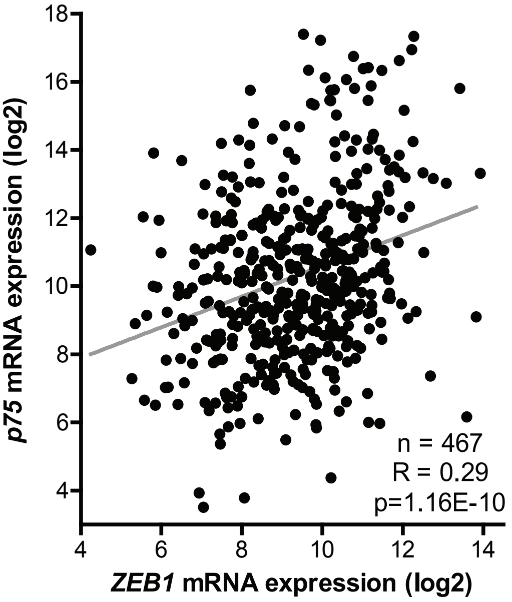

Appendix Figure S6

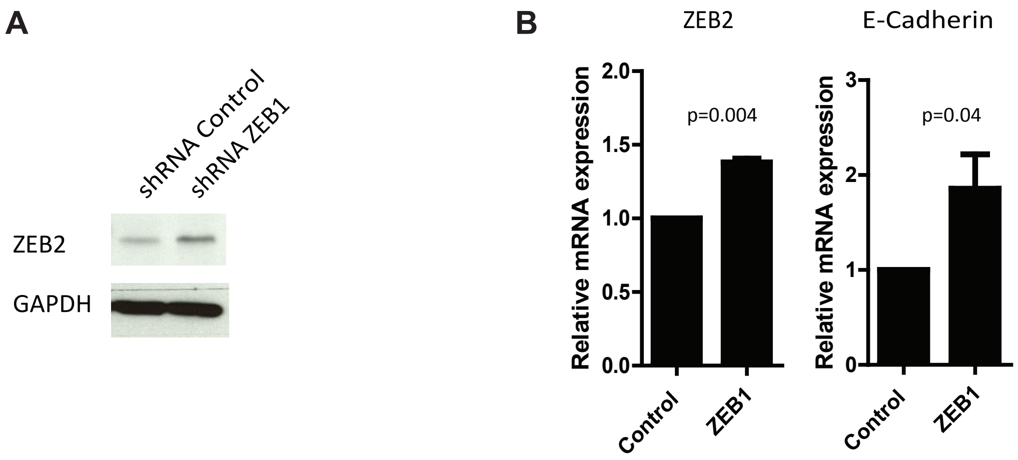

Appendix Figure S7

PLX4032 IC50 (µM)	A375	C-09.10	GLO
Control	0,20	0,11	0,14
ZEB1	0,61	0,18	0,22

Appendix Figure S8

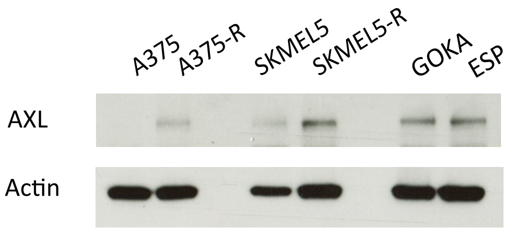

Appendix Figure S9

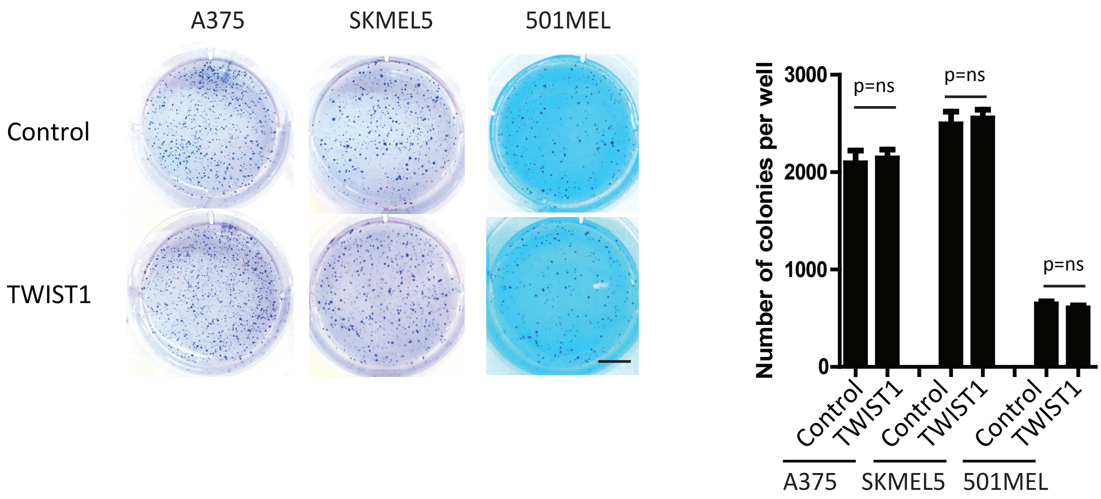

Supplement: Supplementary file 1 — Appendix [file EMMM-8-1143-s001.pdf]
