# Supplementary material for: ZEB1‐mediated melanoma cell plasticity enhances resistance to MAPK inhibitors
Source: EMBO Mol Med. 2016 Sep 5;8(10):1143–61. doi: 10.15252/emmm.201505971 (PMC5048365; doi:10.15252/emmm.201505971)
Supplement: Supplementary file 2 — Expanded View Figures PDF [file EMMM-8-1143-s002.pdf]

Expanded View Figures

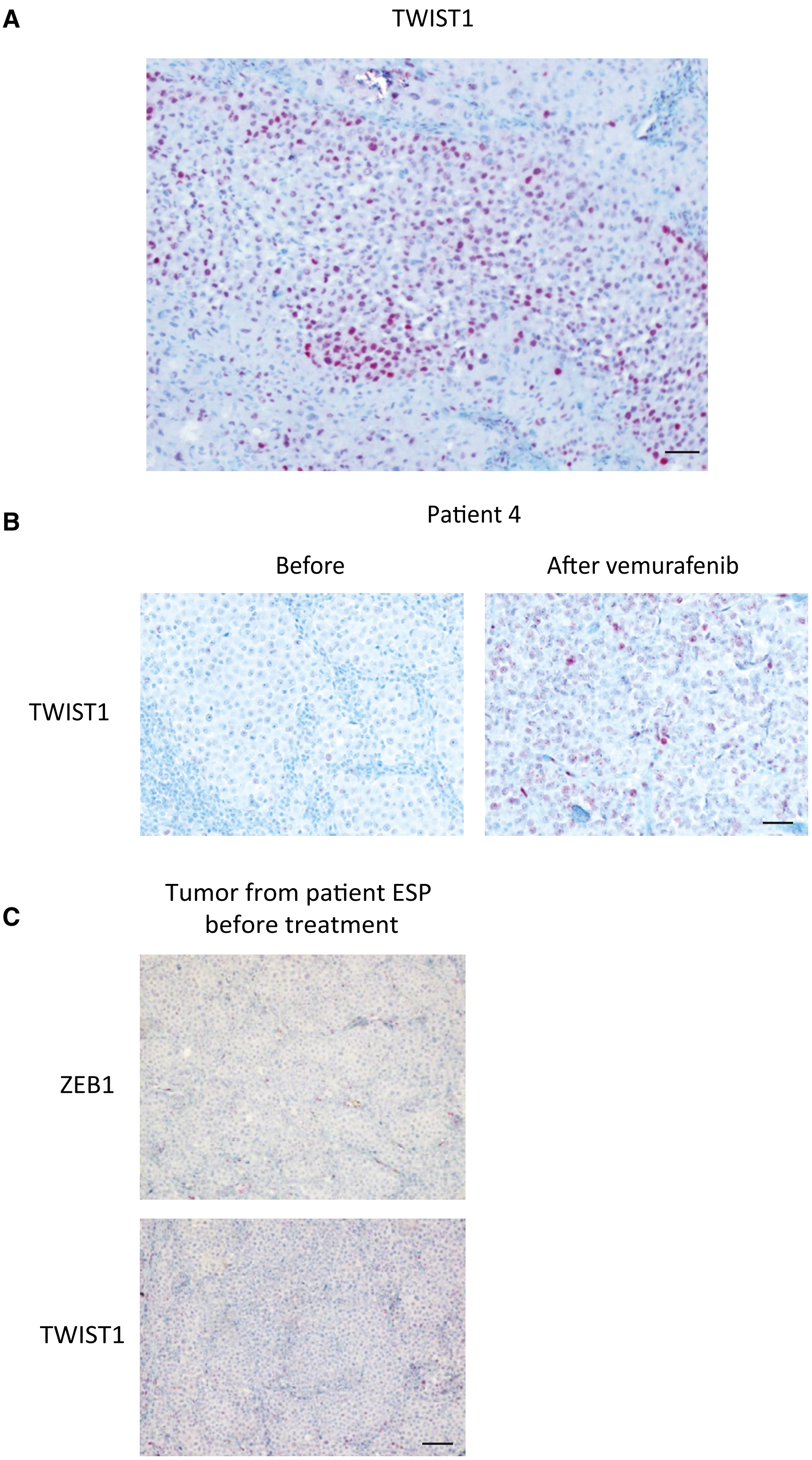

Figure EV1. TWIST1 expression levels are frequently elevated in *BRAF*^{V600} melanoma patients upon vemurafenib treatment.

A Representative picture of TWIST1 immunostaining in the bulk of primary melanoma. Scale bar = 40 µm.

B Representative pictures of TWIST1 immunostaining before and after vemurafenib treatment in patient 4. Scale bar = 40 µm.

C ZEB1 and TWIST1 immunostainings in the tumor from the patient from which ESP cells had been derived, before treatment with vemurafenib. Scale bar = 80 µm.

 © 2016 The Authors

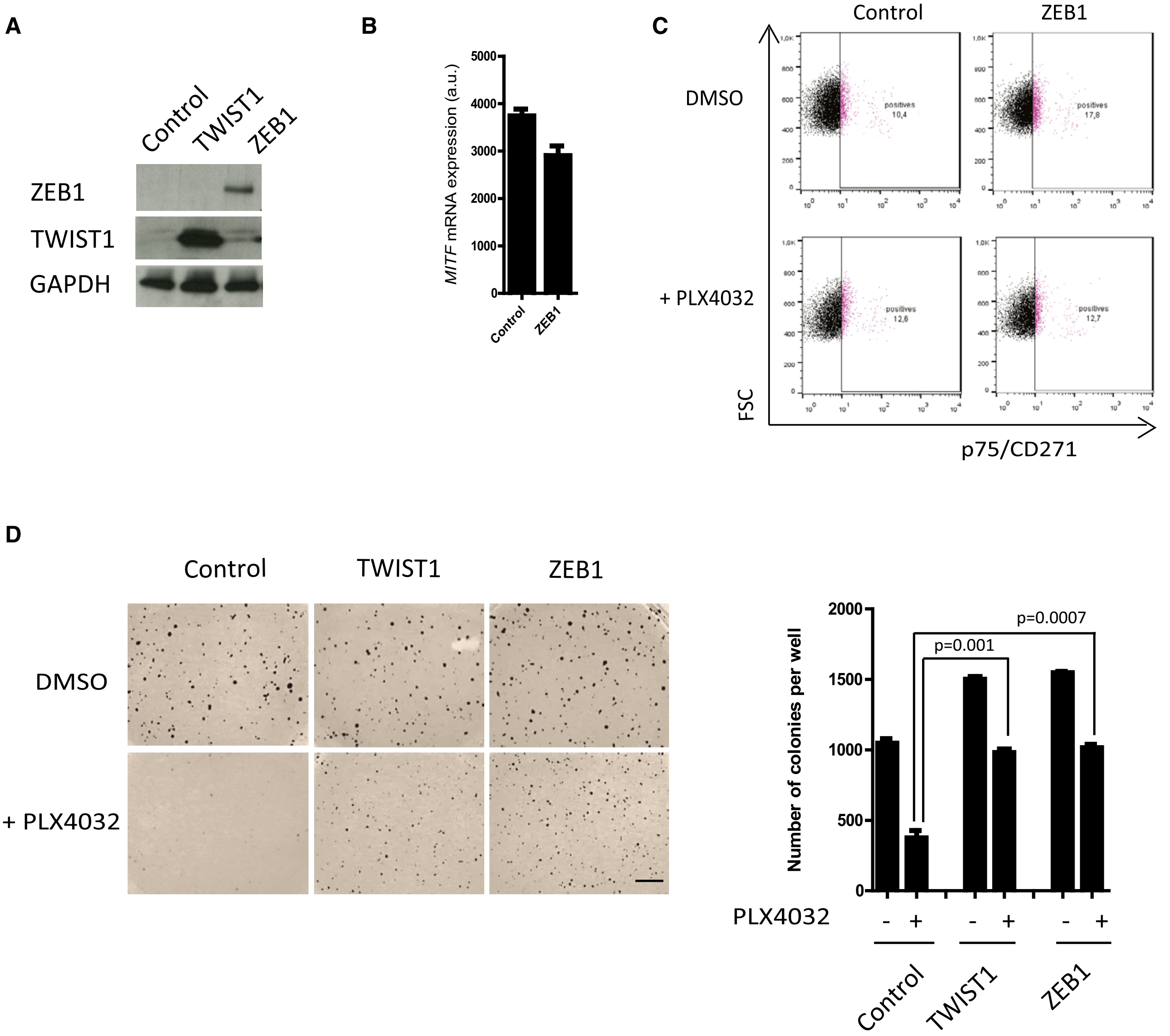

Figure EV2. The ectopic expression of *TWIST1* or *ZEB1* in the ZEB1^{low} 501MEL cell line promotes BRAFi resistance.

A 501MEL cells were infected with retroviruses expressing either *TWIST1* or *ZEB1*. Western blot analyses of ZEB1 and TWIST1. GAPDH was used as a loading control.

B Quantitative PCR analyses of *MITF* expression upon *ZEB1* ectopic expression (mean ± SD, n = 3).

C FACS analyses of p75 expression upon *ZEB1* ectopic expression, after 10 days with or without 100 nM PLX4032 treatment, showing that p75 is not induced in either of these conditions.

D Soft agar colony formation assay in the presence or absence of PLX4032 (100 nM). Scale bar = 500 μm. The graph represents the mean number of colonies (± SD) in three independent experiments (Student's t-test).

Source data are available online for this figure.

© 2016 The Authors

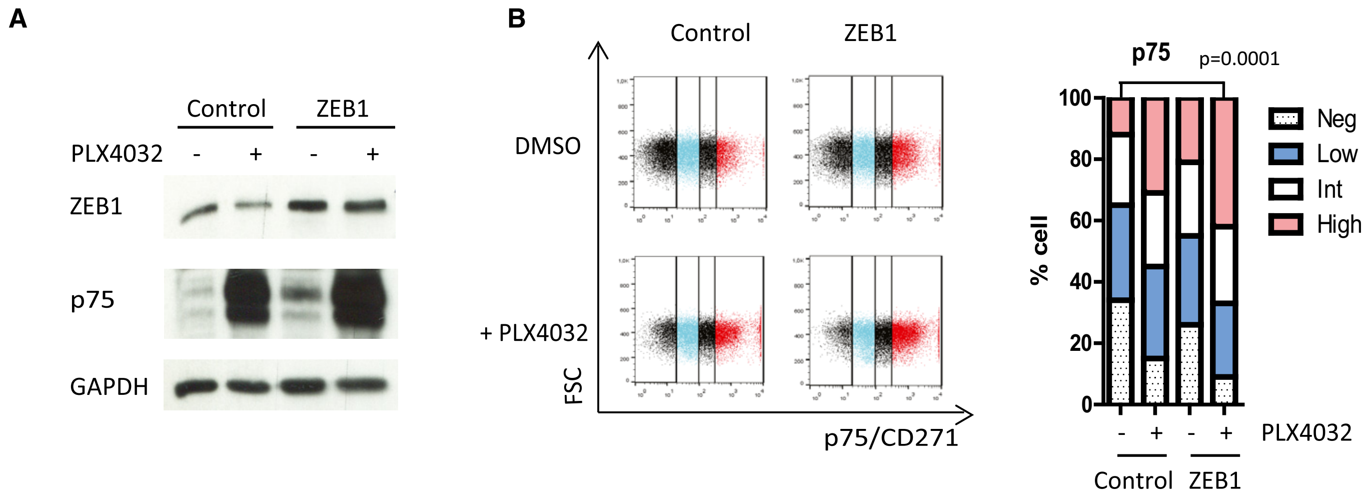

Figure EV3. *ZEB1* ectopic expression in ZEB1^{low} patient-derived short-term culture GLO cells promotes a p75^{high} phenotype.

A GLO short-term culture cells were infected with retroviruses expressing *ZEB1*. Western blot analyses of ZEB1 and p75 in the presence or absence of PLX4032 (100 nM). GAPDH was used as a loading control.

B FACS analyses of p75 expression upon *ZEB1* ectopic expression, after 10 days with or without 100 nM PLX4032 treatment. Bar chart representing the mean percentage of p75^{high}, p75^{int}, p75^{low}, and p75^{negative} cells from two independent experiments (Fisher's exact test).

Source data are available online for this figure.

Figure EV4. *ZEB1* knockdown in SKMEL5 cells promotes a p75^{low} phenotype and sensitizes cells to BRAFi.

A SKMEL5 cells were infected with retroviruses expressing a control or shRNA-*ZEB1*. Western blot analyses of ZEB1 and p75 upon *ZEB1* knockdown. GAPDH was used as a loading control.

B FACS analyses of p75 cell surface expression upon *ZEB1* knockdown. Bar chart representing the mean percentage of p75^{high}, p75^{int}, and p75^{low} cells from two independent experiments.

C Soft agar colony formation assay in the presence or absence of PLX4032 (300 nM). Scale bar = 100 μm. Histograms represent quantitative analyses (mean ± SD, n = 3, Student's t-test).

D 2 × 10^{6} SKMEL5 cells expressing an IPTG-inducible shRNA control or shRNA-*ZEB1* were injected subcutaneously into nude mice. When the tumor reached 5 mm in diameter, ZEB1 expression was silenced by providing mice with IPTG (10 mM) in their drinking water and orally administering vemurafenib (50 mg/kg) on a daily basis for 15 days. The mean tumor volume for five mice is represented (± s.e.m.) (Student's t-test, P at 21 days). ns: nonsignificant.

Source data are available online for this figure.

© 2016 The Authors

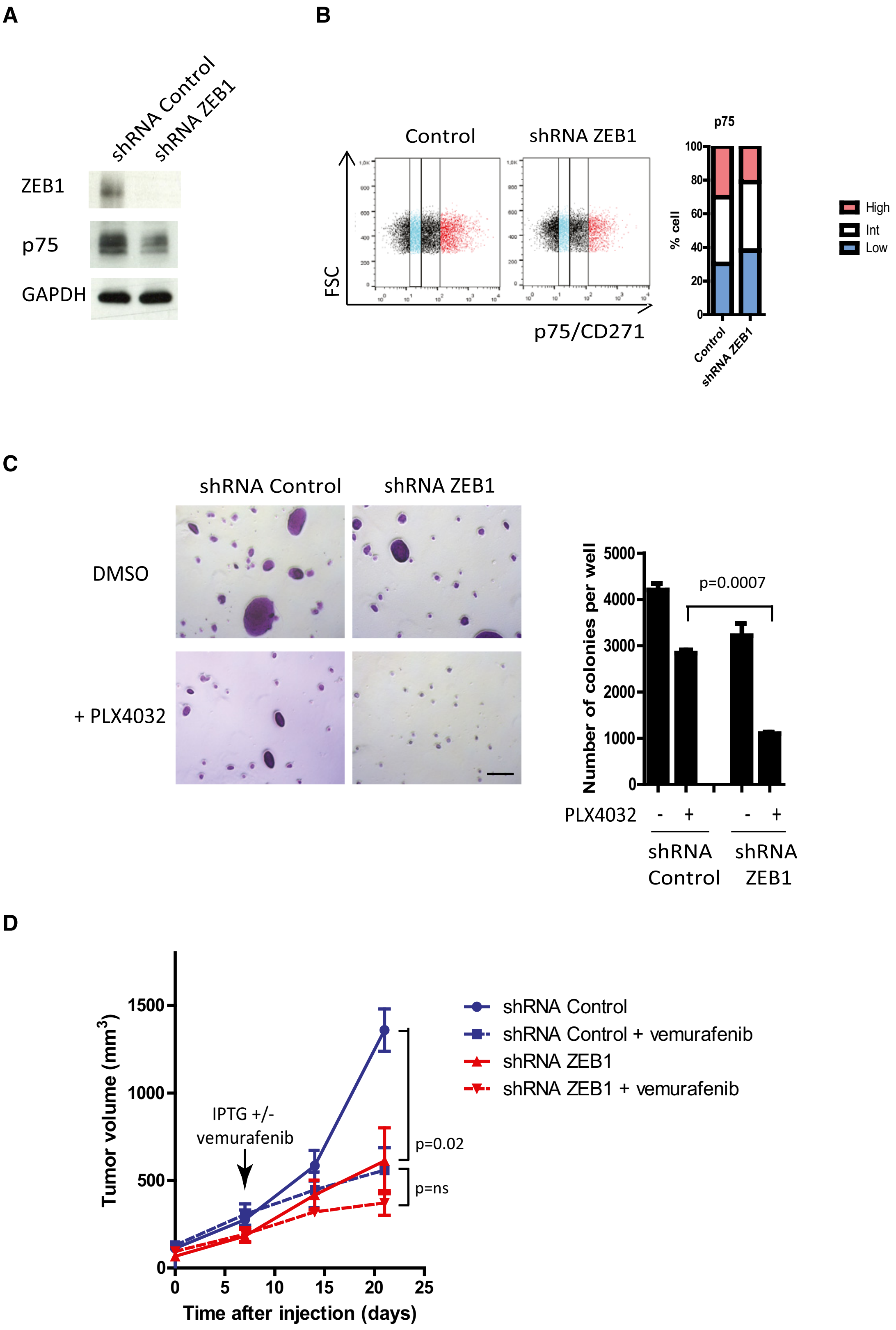
A
shRNA Control
shRNA ZEB1
ZEB1
p75
GAPDH
B
Control
shRNA ZEB1
FSC
p75/CD271
p75
% cell
Control
shRNA ZEB1
High
Int
Low
C
shRNA Control
shRNA ZEB1
DMSO
+ PLX4032
Number of colonies per well
p=0.0007
PLX4032
shRNA Control
shRNA ZEB1
D
Tumor volume (mm³)
IPTG +/- vemurafenib
p=0.02
p=ns
Time after injection (days)
shRNA Control
shRNA Control + vemurafenib
shRNA ZEB1
shRNA ZEB1 + vemurafenib

Figure EV4.
