# Supplementary figures and images for: ZEB1‐mediated melanoma cell plasticity enhances resistance to MAPK inhibitors
Source: EMBO Mol Med. 2016 Sep 5;8(10):1143–61. doi: 10.15252/emmm.201505971 (PMC5048365; doi:10.15252/emmm.201505971)

Source Data Appendix Figure S3

**A**

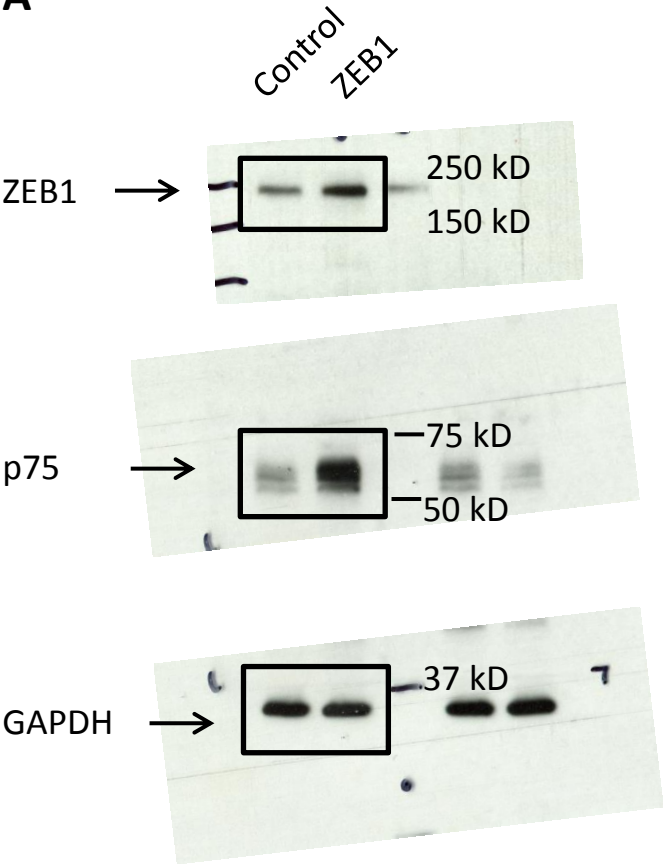

Supplement: Supplementary file 3 — Source Data for Expanded View and Appendix [file EMMM-8-1143-s009.zip › Source_Data_for_Expanded_View_and_Appendix/Source_Data_Figure_Appendix_S3.pdf]

**A**

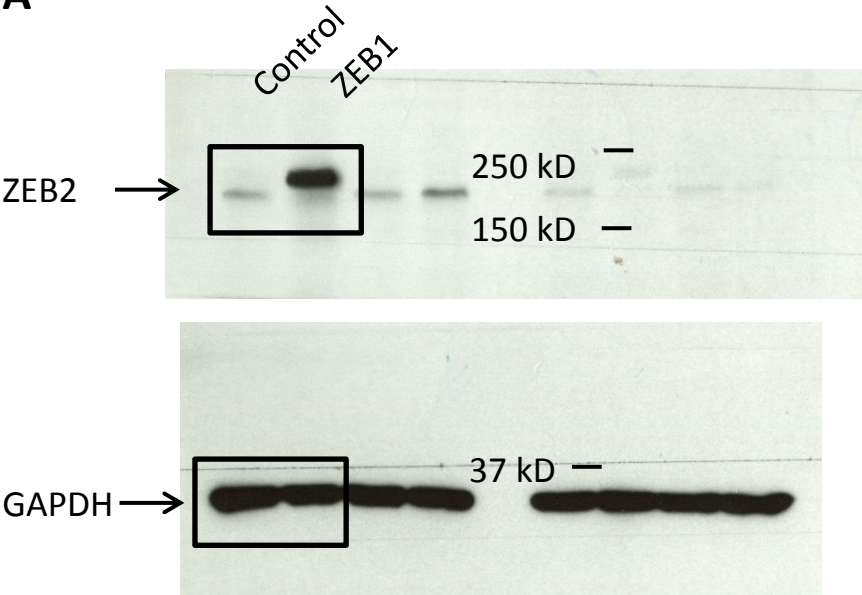

Supplement: Supplementary file 3 — Source Data for Expanded View and Appendix [file EMMM-8-1143-s009.zip › Source_Data_for_Expanded_View_and_Appendix/Source_Data_Figure_Appendix_S4.pdf]

**A**

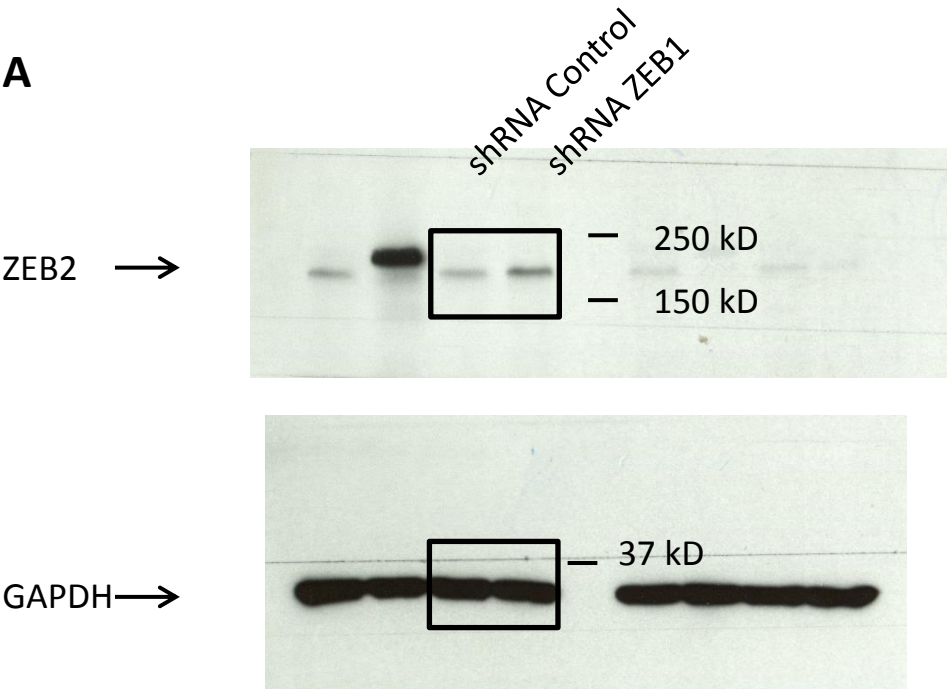

Supplement: Supplementary file 3 — Source Data for Expanded View and Appendix [file EMMM-8-1143-s009.zip › Source_Data_for_Expanded_View_and_Appendix/Source_Data_Figure_Appendix_S6.pdf]

## Source Data Appendix Figure S8

**A**

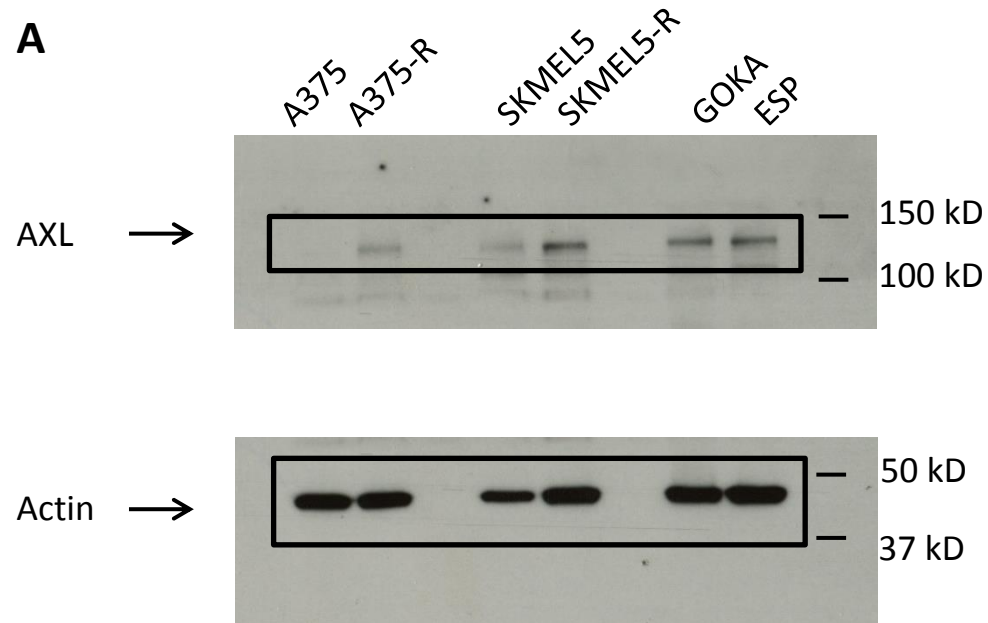

Supplement: Supplementary file 3 — Source Data for Expanded View and Appendix [file EMMM-8-1143-s009.zip › Source_Data_for_Expanded_View_and_Appendix/Source_Data_Figure_Appendix_S8.pdf]

Source Data Figure EV2

**A**

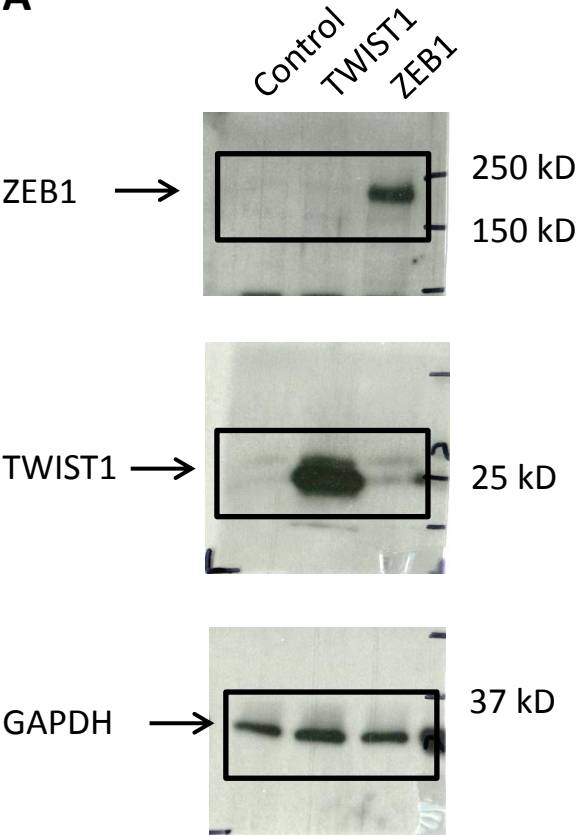

Supplement: Supplementary file 3 — Source Data for Expanded View and Appendix [file EMMM-8-1143-s009.zip › Source_Data_for_Expanded_View_and_Appendix/Source_Data_Figure_EV2.pdf]

Source Data Figure EV3

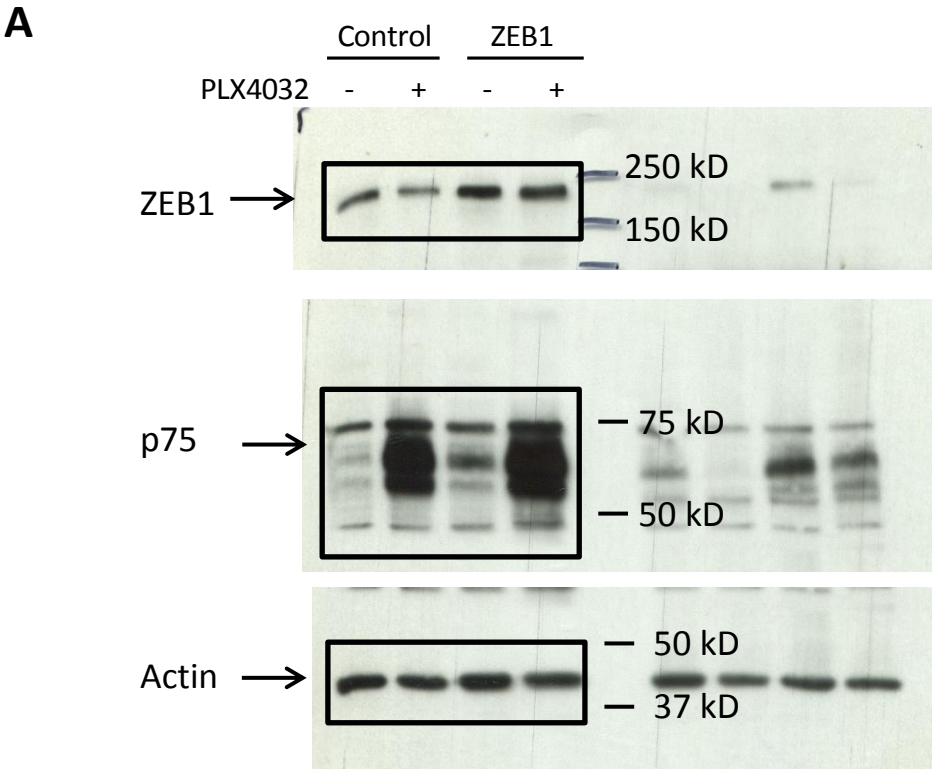

Supplement: Supplementary file 3 — Source Data for Expanded View and Appendix [file EMMM-8-1143-s009.zip › Source_Data_for_Expanded_View_and_Appendix/Source_Data_Figure_EV3.pdf]

Source Data Figure EV4

A

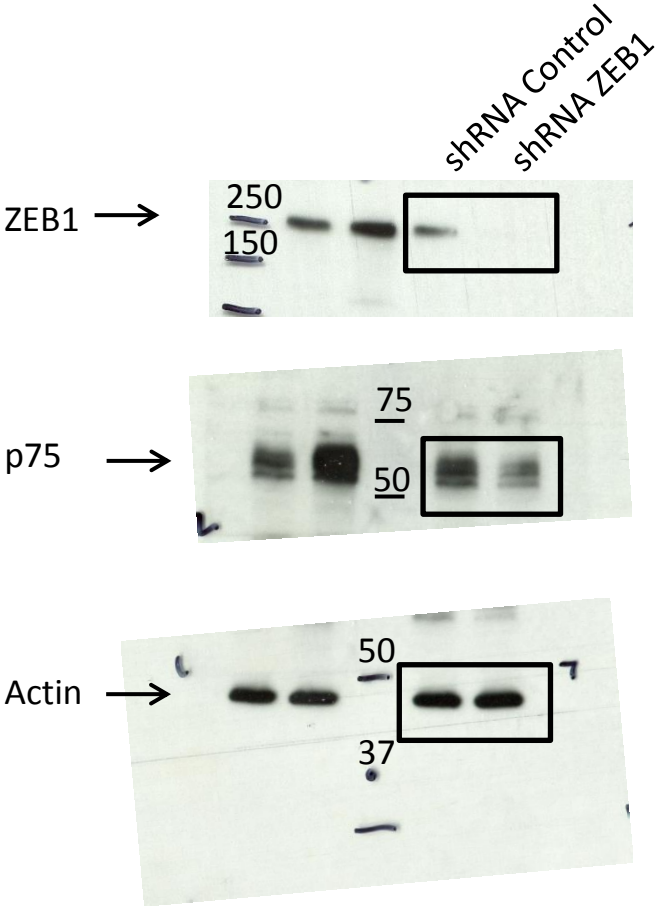

Supplement: Supplementary file 3 — Source Data for Expanded View and Appendix [file EMMM-8-1143-s009.zip › Source_Data_for_Expanded_View_and_Appendix/Source_Data_Figure_EV4.pdf]

Source Data Figure 1

**B**

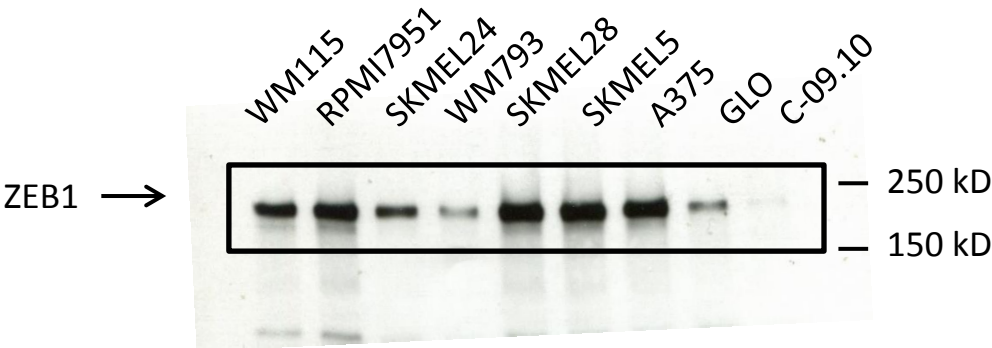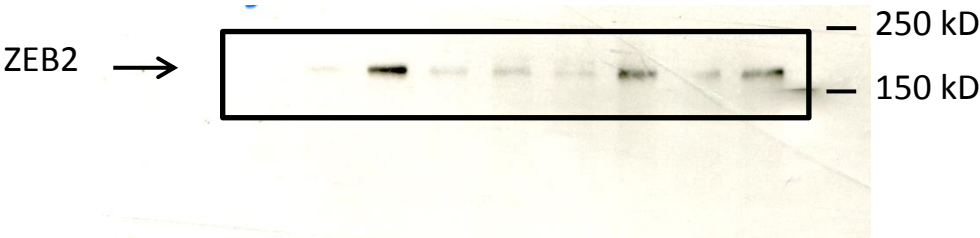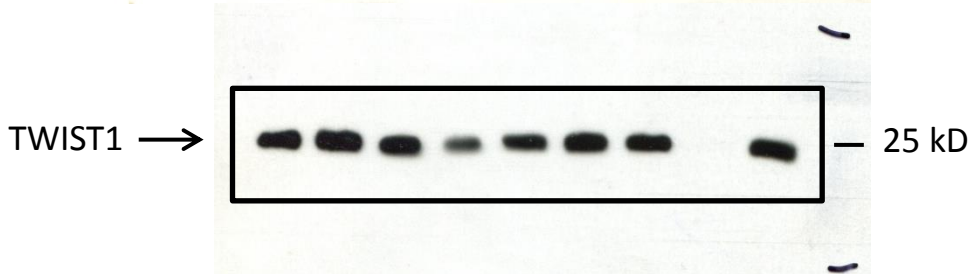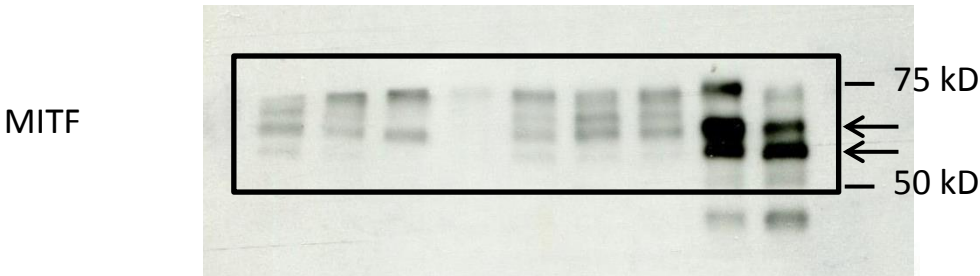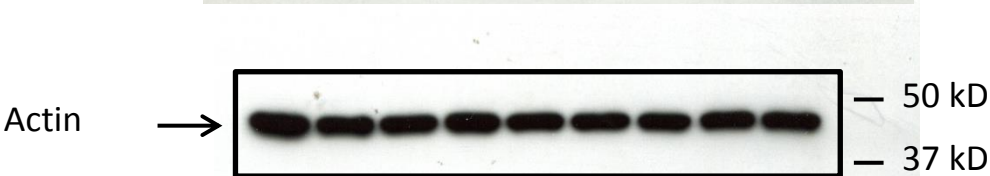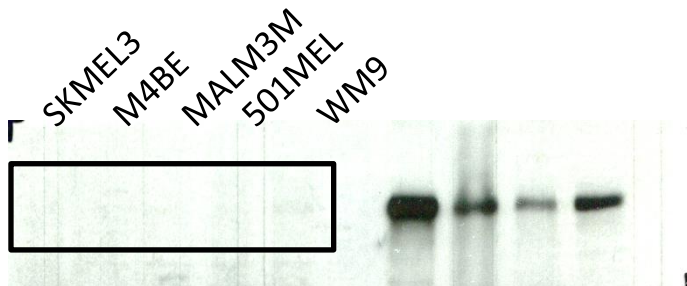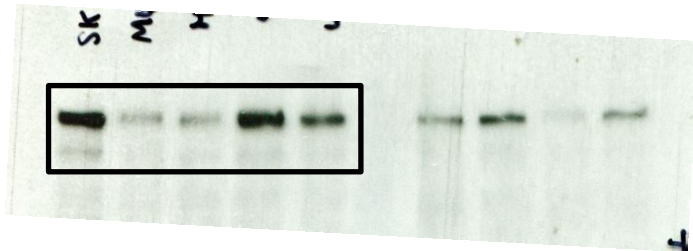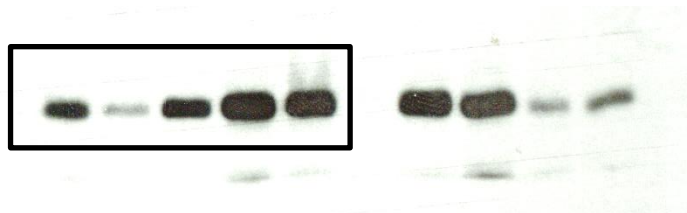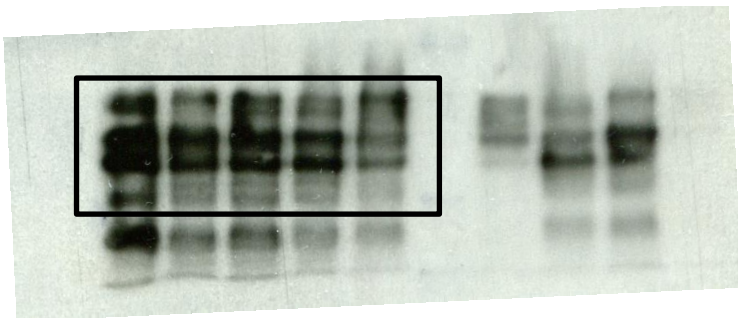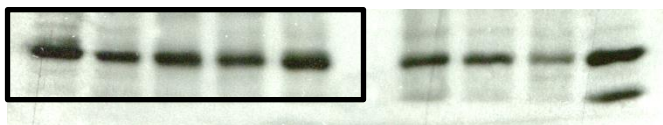

Supplement: Supplementary file 5 — Source Data for Figure 1 [file EMMM-8-1143-s003.pdf]

Source Data Figure 3

**B**

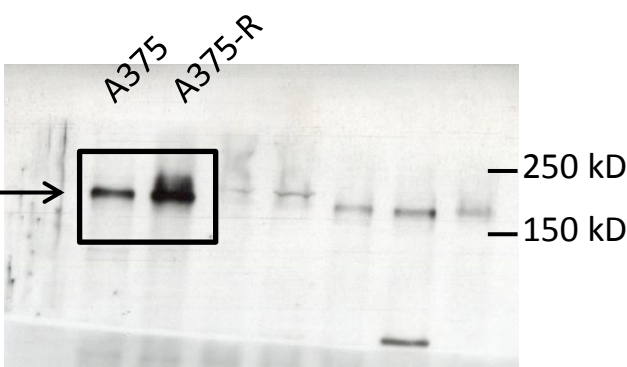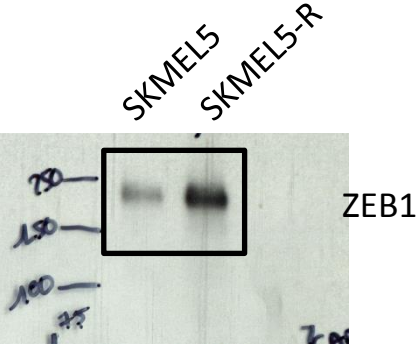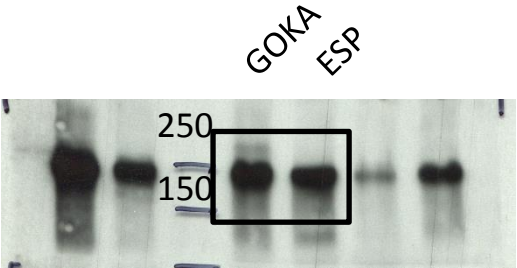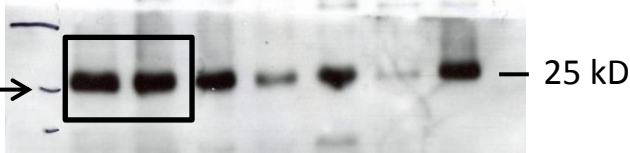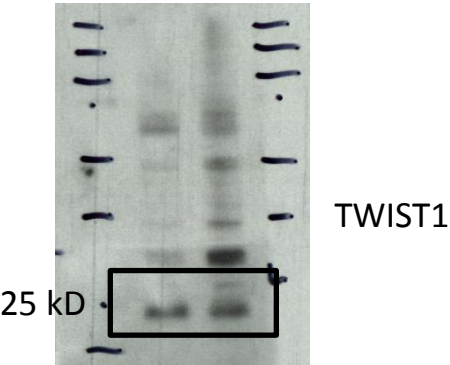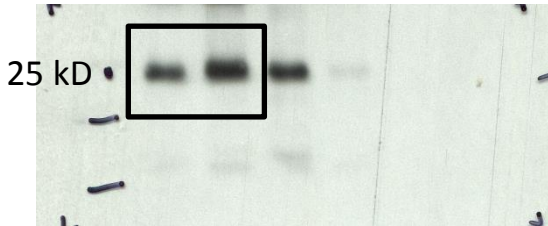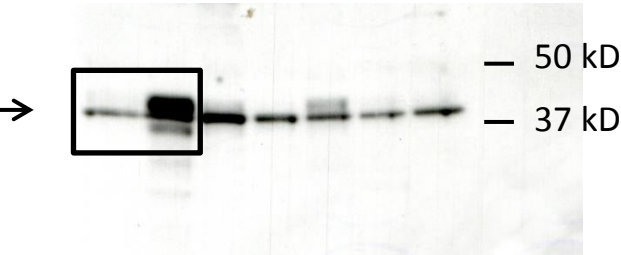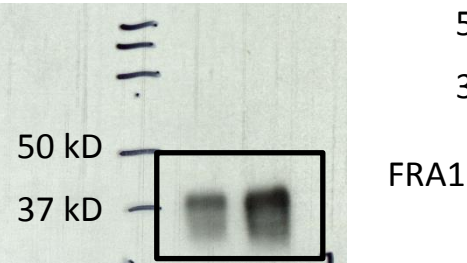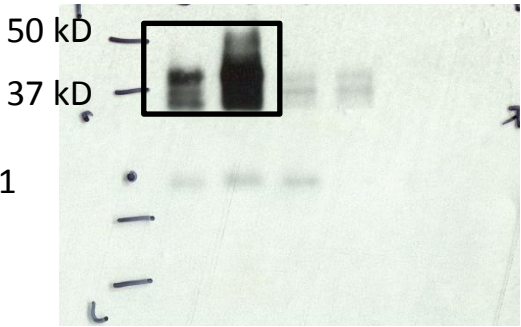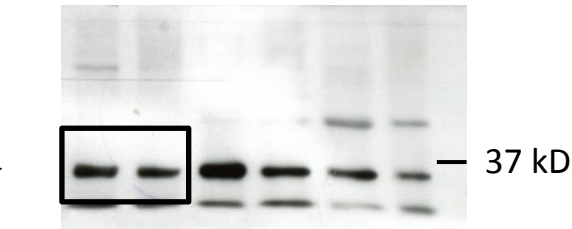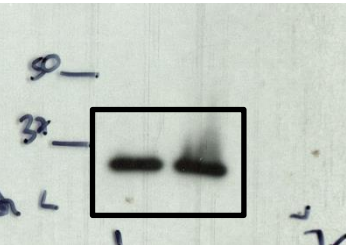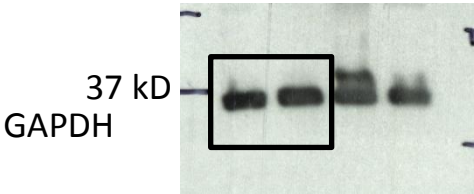

Supplement: Supplementary file 6 — Source Data for Figure 3 [file EMMM-8-1143-s004.pdf]

Source Data Figure 4

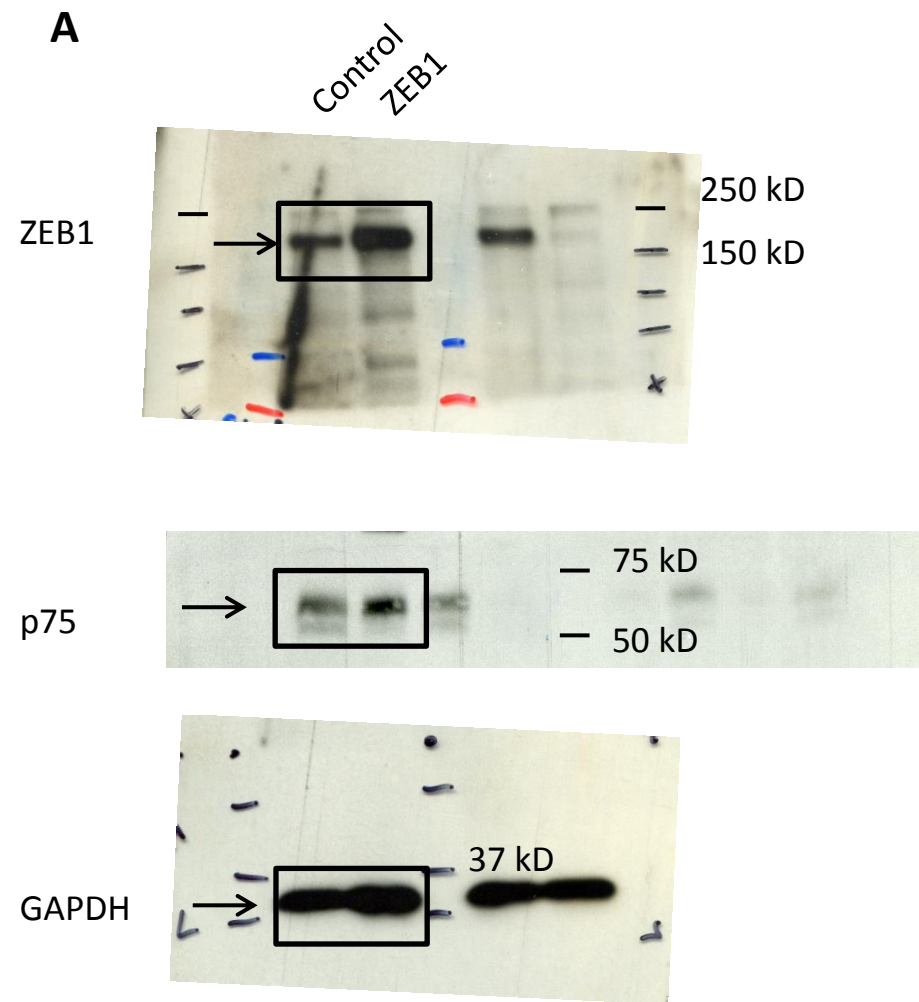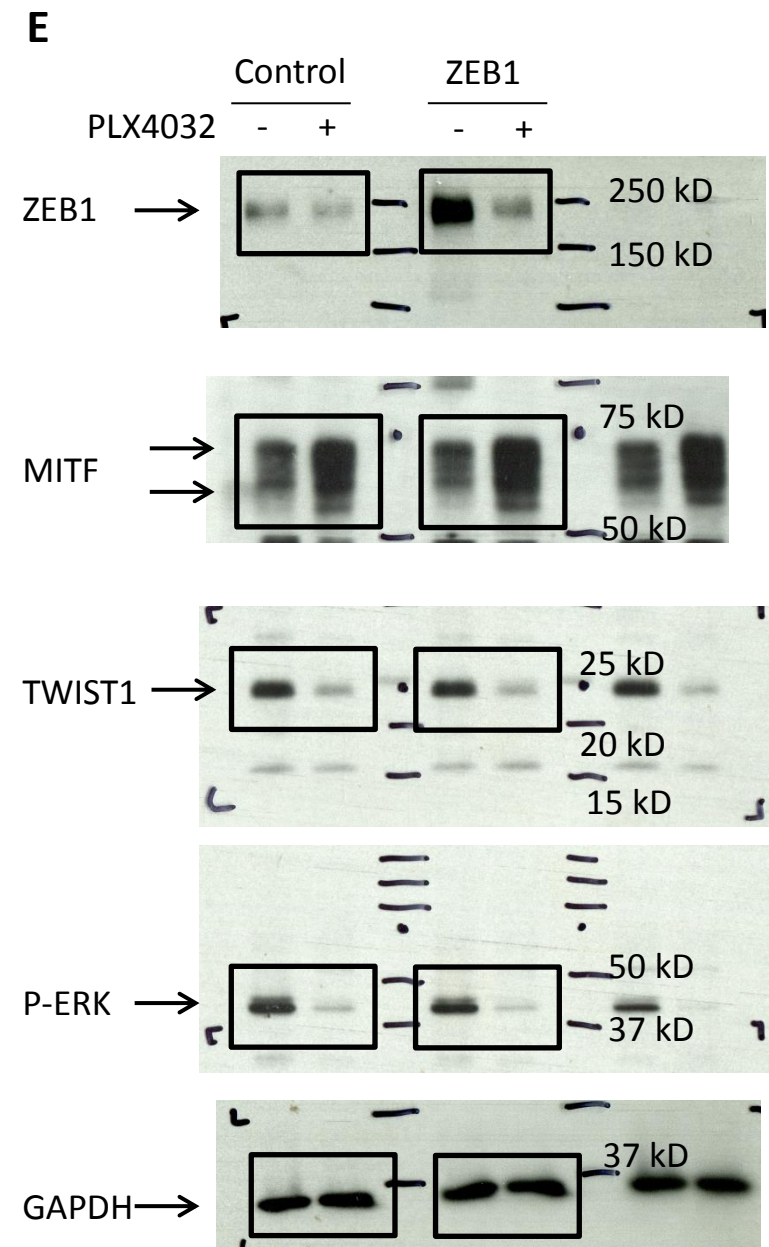

Supplement: Supplementary file 7 — Source Data for Figure 4 [file EMMM-8-1143-s005.pdf]

Source Data Figure 5

**A**

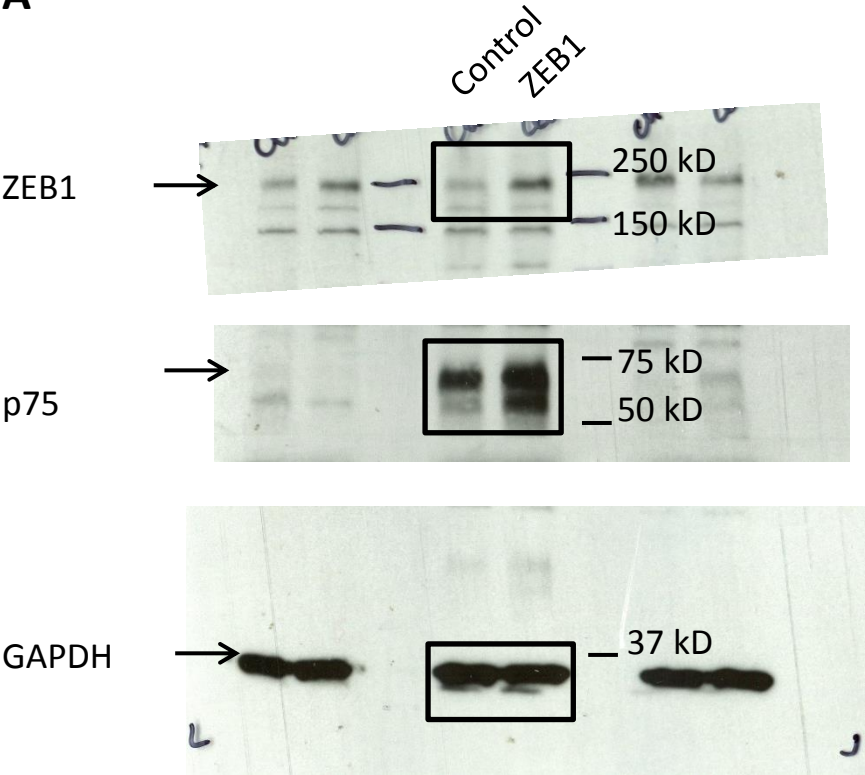

Supplement: Supplementary file 8 — Source Data for Figure 5 [file EMMM-8-1143-s006.pdf]

Source Data Figure 6

**A**

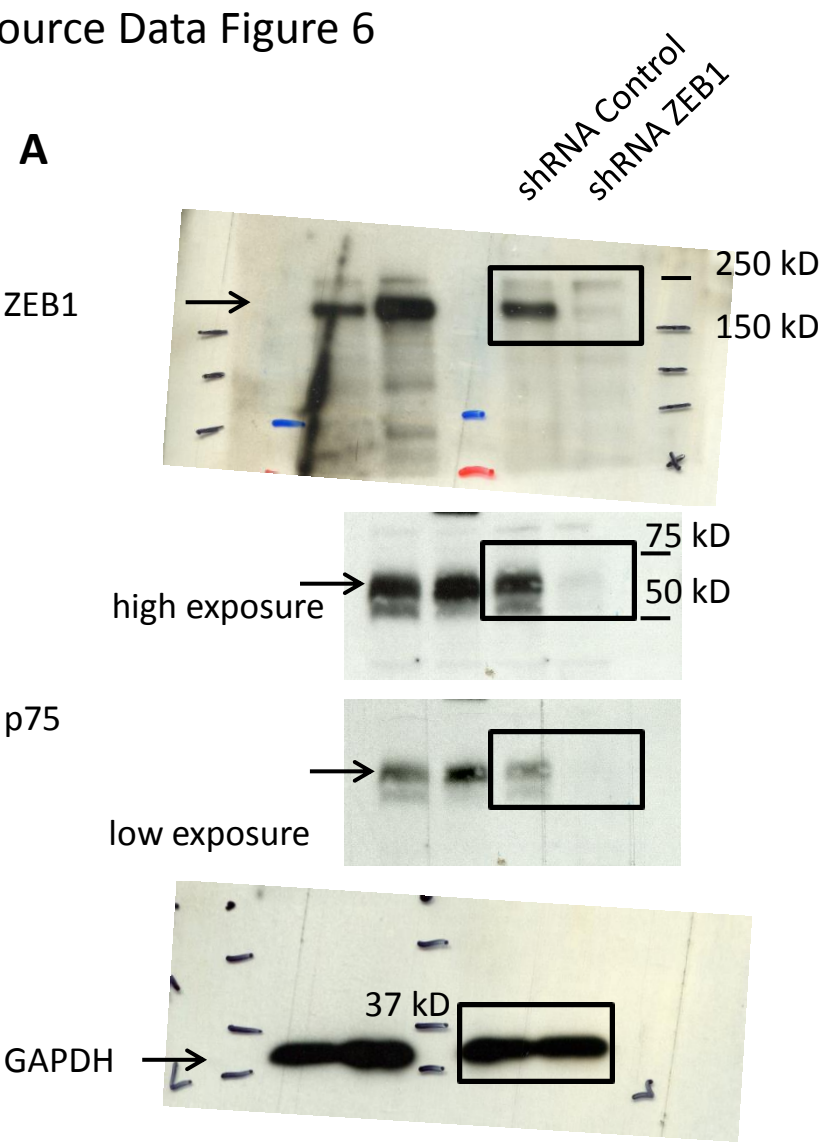

**F**

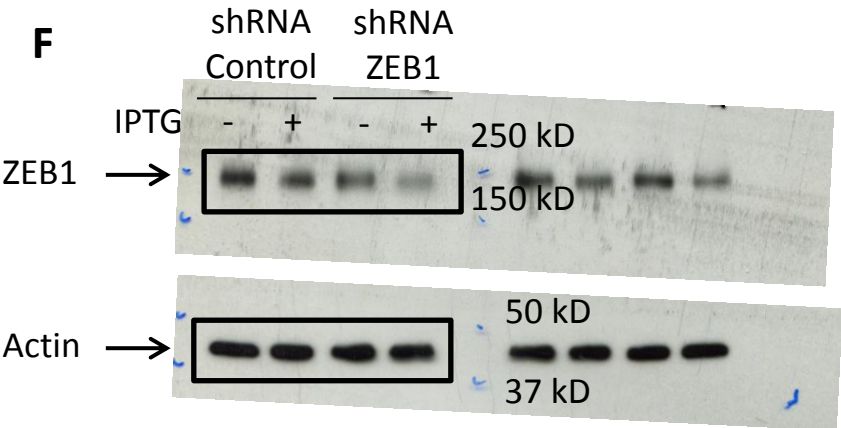

**G**

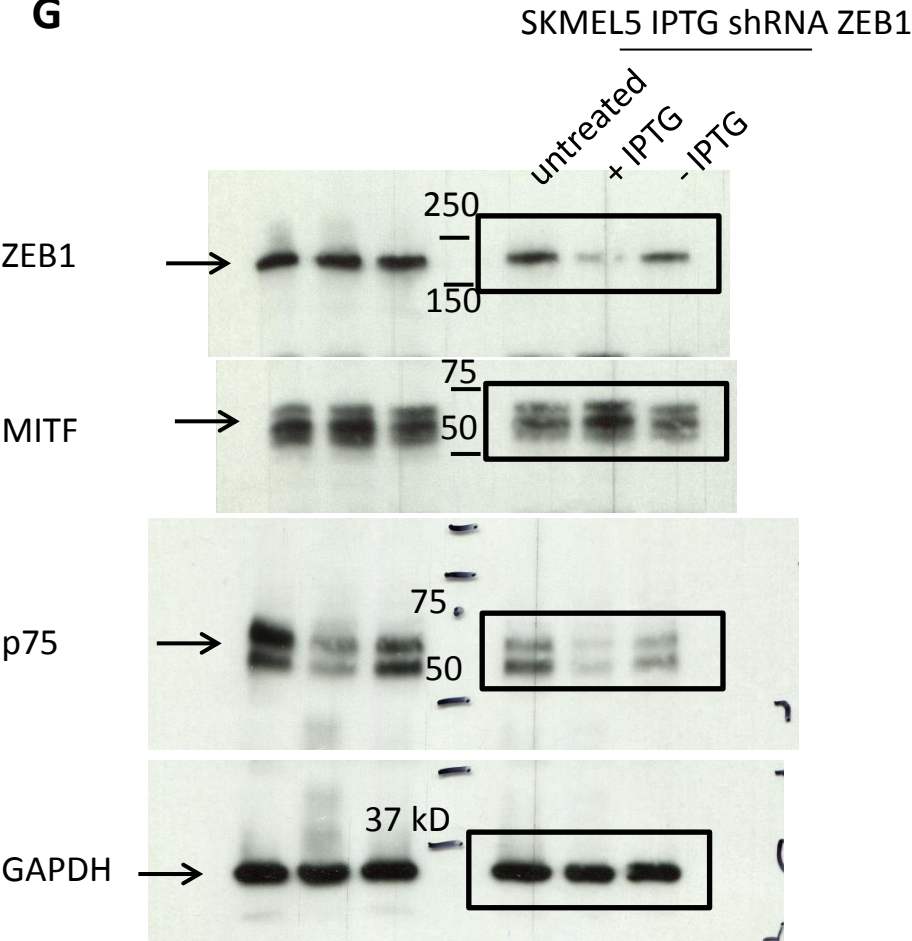

Supplement: Supplementary file 9 — Source Data for Figure 6 [file EMMM-8-1143-s007.pdf]

Source Data Figure 7

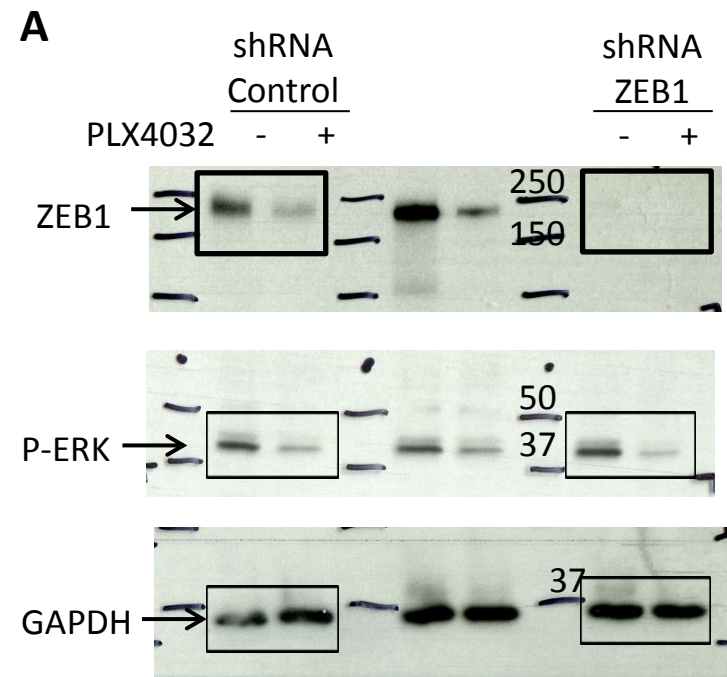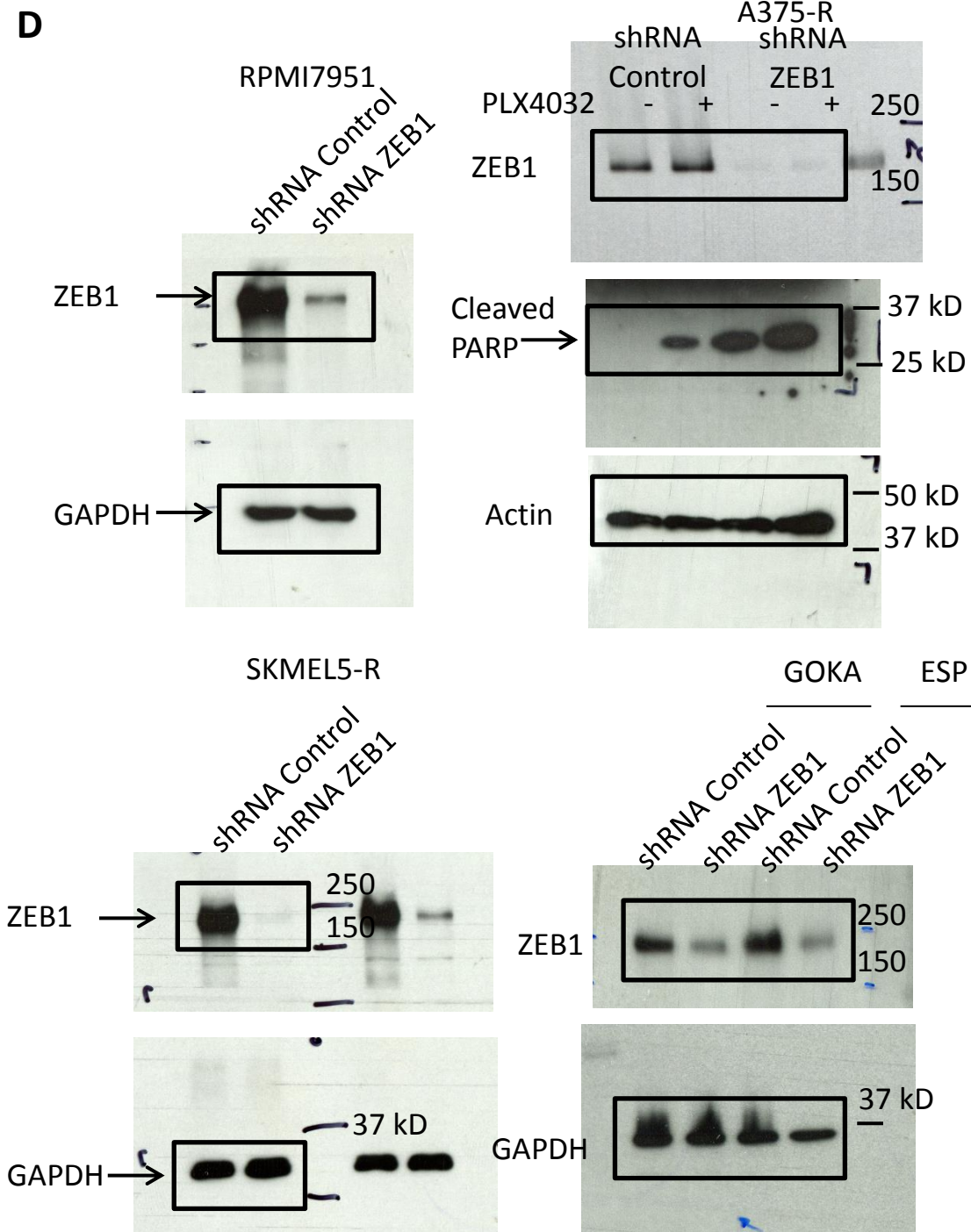

Figure 7

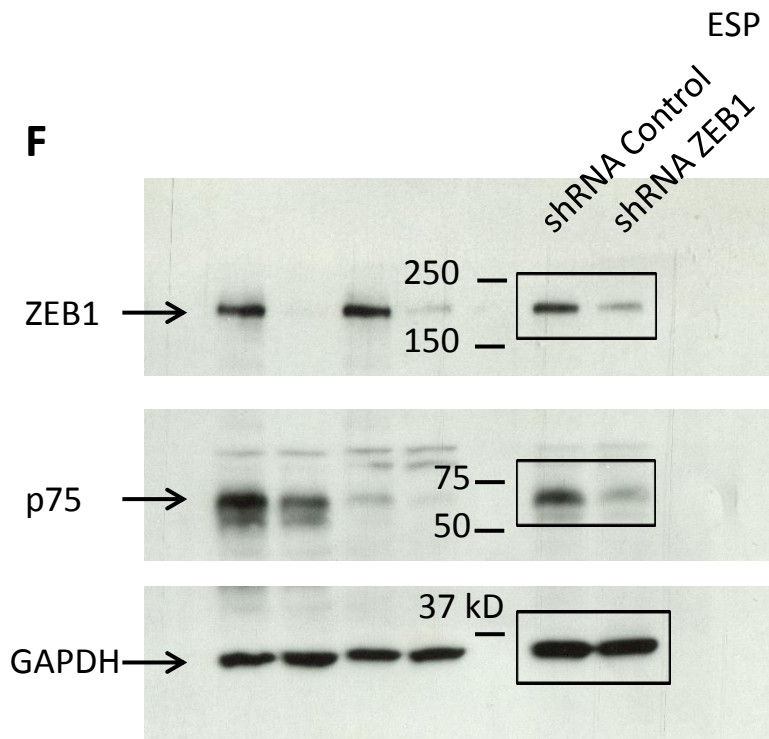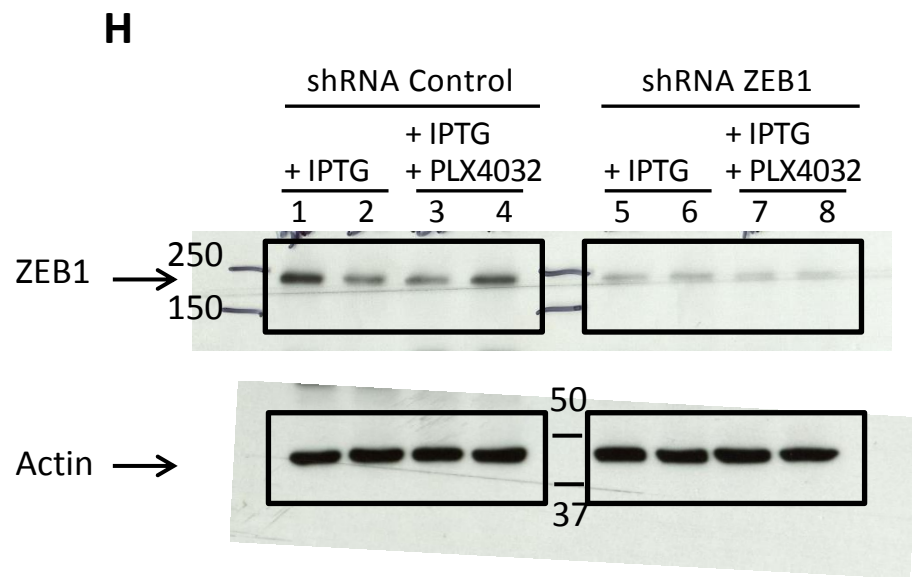

Supplement: Supplementary file 10 — Source Data for Figure 7 [file EMMM-8-1143-s008.pdf]
